# Long-Lasting Changes in Glial Cells Isolated From Rats Subjected to the Valproic Acid Model of Autism Spectrum Disorder

**DOI:** 10.3389/fphar.2021.707859

**Published:** 2021-08-05

**Authors:** Marianela Evelyn Traetta, Nonthué Alejandra Uccelli, Sandra Cristina Zárate, Dante Gómez Cuautle, Alberto Javier Ramos, Analía Reinés

**Affiliations:** ^1^ Instituto de Biología Celular y Neurociencia “Prof. E. De Robertis” (IBCN), CONICET - Universidad de Buenos Aires, Buenos Aires, Argentina; ^2^ Facultad de Farmacia y Bioquímica, Cátedra de Farmacología, Universidad de Buenos Aires, Buenos Aires, Argentina; ^3^ Facultad de Medicina, Departamento de Histología, Embriología, Biología Celular y Genética, Universidad de Buenos Aires, Buenos Aires, Argentina; ^4^ Instituto de Investigaciones Biomédicas (INBIOMED), CONICET - Universidad de Buenos Aires, Buenos Aires, Argentina

**Keywords:** neuroinflammation, microglia, astrocyte, synapse, autism

## Abstract

Synaptic alterations concomitant with neuroinflammation have been described in patients and experimental models of autism spectrum disorder (ASD). However, the role of microglia and astroglia in relation to synaptic changes is poorly understood. Male Wistar rats prenatally exposed to valproic acid (VPA, 450 mg/kg, i.p.) or saline (control) at embryonic day 10.5 were used to study synapses, microglia, and astroglia in the prefrontal cortex (PFC) at postnatal days 3 and 35 (PND3 and PND35). Primary cultures of cortical neurons, microglia, and astroglia isolated from control and VPA animals were used to study each cell type individually, neuron-microglia and microglia-astroglia crosstalk. In the PFC of VPA rats, synaptic changes characterized by an increase in the number of excitatory synapses were evidenced at PND3 and persisted until PND35. At PND3, microglia and astroglia from VPA animals were morphologically similar to those of age-matched controls, whereas at PND35, reactive microgliosis and astrogliosis were observed in the PFC of VPA animals. Cortical neurons isolated from VPA rats mimicked *in vitro* the synaptic pattern seen *in vivo*. Cortical microglia and astroglia isolated from VPA animals exhibited reactive morphology, increased pro-inflammatory cytokines, and a compromised miRNA processing machinery. Microglia from VPA animals also showed resistance to a phagocytic challenge. In the presence of neurons from VPA animals, microglia isolated from VPA rats revealed a non-reactive morphology and promoted neurite outgrowth, while microglia from control animals displayed a reactive profile and promoted dendritic retraction. In microglia-astroglia co-cultures, microglia from VPA animals displayed a reactive profile and exacerbated astrocyte reactivity. Our study indicates that cortical microglia from VPA animals are insensitive or adapted to neuronal cues expressed by neurons from VPA animals. Further, long-term *in vivo* microgliosis could be the result of altered microglia-astroglia crosstalk in VPA animals. Thus, our study highlights cortical microglia-astroglia communication as a new mechanism implicated in neuroinflammation in ASD; consequently, we propose that this crosstalk is a potential target for interventions in this disorder.

## Introduction

Autism spectrum disorder (ASD) is a developmental condition characterized by social and communication deficits and restricted, repetitive patterns of behavior, interests, or activities (American Psychiatric Association, 2013). ASD neurobiology includes genetic and environmental factors (De Rubeis et al., 2014; Modabbernia et al., 2017). The genetic basis is complex, and alterations in epigenetic pathways have also been reported (De Rubeis et al., 2014; Geschwind and Flint, 2015). Several large-scale genetic studies have highlighted many genes as risk factors for ASD pathogenesis (Sebat et al., 2007; Anney et al., 2010; De Rubeis et al., 2014; Iossifov et al., 2014). Particularly, point mutations, gene deletions, and polymorphisms for several synaptic components such as cell adhesion molecules, scaffold proteins, and glutamate receptors have been documented (Betancur et al., 2009; Leblond et al., 2014; Hu et al., 2016). Moreover, dendritic alterations that might affect synapse formation and stability and altered spine density were observed in different brain areas in patients with ASD (Raymond et al., 1996; Hutsler and Zhang, 2010). Given that affected genes are linked to synaptic function and plasticity (Bourgeron, 2015; Lin et al., 2016) and structural dendritic and synaptic alterations were described in ASD, this disorder has been proposed to be a synaptopathy (Guang et al., 2018).

Neuroinflammation has been extensively documented in ASD, described as microgliosis and astrogliosis in different brain regions and increased expression of pro-inflammatory cytokines (Liao et al., 2020). Both astrocytes and microglia functions are critical for proper brain development: microglia promote synapse formation and refine circuits by pruning synapses (Stevens et al., 2007; Miyamoto et al., 2016), and astrocytes are critical in maintaining physiological homeostasis within the brain, playing important roles in synapse formation and supporting neuronal function (Araque and Navarrete, 2010; Allen and Eroglu, 2017). Both glial cells impact and react to the environment, making their contribution a valuable target to modulate (Zhang et al., 2010; Lenz and Nelson, 2018). Indeed, it is still a matter of debate whether glial alterations during development lead to ASD onset or neuroinflammation is a pathophysiological response to altered synaptic connectivity.

During neuroinflammation, microglia are the first glial cells to react by releasing cytokines that engage astrocytes, further intensifying neuroinflammation (Zhang et al., 2010; Kirkley et al., 2017; Liddelow et al., 2017; Auzmendi et al., 2020). Microglia rapidly adapt to the environment by changing their morphology: they shift from a ramified morphology to an amoeboid shape by retracting and thickening their processes and enlarging their soma (Kreutzberg, 1996; Matta et al., 2019). It is worth mentioning that prolonged dysregulated microglial activity has been implicated in brain neurotoxicity (Dheen et al., 2007). Astrocyte reactive gliosis is characterized by upregulation of intermediate filaments, particularly glial fibrillary acidic protein (GFAP) (Hol and Pekny, 2015) and morphological changes characterized by process extension and hypertrophy (Sofroniew and Vinters, 2010). There is also a bidirectional communication between microglia and astrocytes mediated by the increased secretion of signaling molecules and cytokines (Zhang et al., 2010; Matta et al., 2019; Matejuk and Ransohoff, 2020). For instance, reactive microglia release of IL1, IL6, and TNFα can trigger astrocyte reactivity (Zhang et al., 2010; Liddelow et al., 2017). In turn, reactive astrocytes release ATP and promote microglia activation (Coco et al., 2003; Bianco et al., 2005; Davalos et al., 2005).

Idiopathic ASD can be modeled in rats by prenatal exposure to valproic acid (VPA). This experimental model (VPA model) mimics the main behavioral and neuroanatomical alterations found in patients with ASD (Schneider and Przewłocki, 2005; Roullet et al., 2013; Nicolini and Fahnestock, 2018). Both neuronal and glial changes have been described in different brain regions in this model (Codagnone et al., 2015; Bronzuoli et al., 2018; Nicolini and Fahnestock, 2018; Traetta et al., 2021). Indeed, we have described changes in synaptic markers in key areas related to ASD behavioral deficits, such as the prefrontal cortex (PFC) and the hippocampus (Codagnone et al., 2015; Traetta et al., 2021). In particular, the PFC plays a key role in social behavior, decision-making, and emotional processing (Dixon et al., 2017; Chini and Hanganu-Opatz, 2021). In VPA animals, the PFC is characterized by synaptic changes shown as an increase in the synaptic marker synaptophysin (SYN) (Codagnone et al., 2015) and hyper-connectivity and hyper-plasticity (Rinaldi et al., 2008). It is worth mentioning that these synaptic changes are shown concomitantly with microgliosis and astrogliosis (Codagnone et al., 2015; Bronzuoli et al., 2018). Thus, this brain region is a key area to study the role of microglia and astroglia in ASD. Defining the contribution of these glial cells in the context of synaptic alterations may provide new targets for modulating neuroinflammation in ASD.

The present study explores the role of microgliosis and astrogliosis in relation to the synaptic changes in ASD. Therefore, we focused on cortical synapses, microglia, and astroglia using the rat VPA model. We used an *in vivo* experimental design to shed light on the temporal association of cortical synaptic and glial changes and an *in vitro* approach to study neuronal, microglia, and astroglia individually and neuron-microglia and microglia-astroglia crosstalk. Our work shows that microglia and astroglia display long-lasting changes and a distinctive pro-inflammatory program *in vitro*, altering not only the cytokine profile but also miRNA processing machinery. While microglia from VPA animals are adapted to their neuronal substrate, microglia-astroglia crosstalk exacerbates reactivity. We provide evidence that cortical microglia are adapted to VPA-induced neuronal alterations and *in vivo* gliosis could result from altered microglia-astroglia crosstalk. Therefore, our study highlights the critical role of microglia-astroglia communication as a new mechanism underlying neuroinflammation in ASD and suggests this process as a new target for interventions.

## Materials and Methods

### Animals

The VPA model was induced as previously described (Schneider and Przewłocki, 2005; Traetta et al., 2021). Wistar rats provided by Facultad de Ciencias Exactas y Naturales (Universidad de Buenos Aires) were housed in an air-conditioned room (20 ± 2°C) and maintained on a 12 h light/dark cycle with food and water *ad libitum.* They were mated overnight, and the day when spermatozoa were found in vaginal smears was considered embryonic day (E) 0. On E10.5, pregnant dams were given 450 mg/kg VPA intraperitoneally (sodium valproate, Parafarm, Droguería Saporiti S.A.C.I.F.I.A.) or saline solution. Experiments in this study were performed on male pups only: those prenatally exposed to VPA were VPA animals and those exposed to saline were control animals. Pups used for neuronal and glial cultures were born from different dams: each primary neuronal culture was obtained from 3 control and 3 VPA animals at postnatal day (PND) 1 and each primary glial culture from 4 control and 4 VPA animals at PND3. Male siblings were used for evaluating early postnatal development and juvenile behavior to validate the model (Supplementary Materials and Methods). At PND3 and PND35, 4–6 control and 4–6 VPA animals in each PND from 4 different saline and 4 VPA-injected dams were used for immunofluorescence assays; 4 animals (PND35) per group from 3 different saline and 3 VPA-injected dams were used for the electron microscopy study. All experiments were carried out following the Guide for the Care and Use of Laboratory Animals provided by the NIH (United States) and were approved by the Ethics Committee for the Care and Use of Laboratory Animals of the School of Pharmacy and Biochemistry at Universidad de Buenos Aires (Approval No. 180613–1 and 2320).

### Primary Cortical Cultures

***Primary cortical neuronal culture:*** cortical neuronal cultures were prepared from PND1 control and VPA animals as previously described (Reinés et al., 2012; Traetta et al., 2021). The entire cerebral cortex was used. Cells were plated at a density of 5 × 10^4^ cells/cm^2^ on poly-d-lysine (Sigma-Aldrich Co.) coated glass coverslips for immunofluorescence assays. They were maintained for up to 7 days *in vitro* (DIV) in a Neurobasal Medium supplemented with 2% v/v B27 and 0.5 mM glutamine (All from Gibco, Invitrogen Carlsbad, CA, United States).

***Primary cortical glial cultures and treatments:*** primary mixed cortical glial cultures were prepared from control and VPA animals at PND3 as previously described (Rosciszewski et al., 2019; Zárate et al., 2019; Traetta et al., 2021). The entire cerebral cortex was used. Once cells reached confluence, they were subjected to shaking at 180 rpm for 24 h; microglia cells were detached and re-seeded on poly-d-lysine-coated glass coverslips at a density of 4 × 10^4^ cells/cm^2^ for treatments and immunofluorescence assays or at a density of 6 × 10^4^ cells/cm^2^ on plastic dishes for flow cytometry. They were kept up to 7 DIV in Dulbecco’s Modified Eagle Medium (DMEM; Gibco, Invitrogen Carlsbad, CA, United States) supplemented with 50% v/v F12 (Gibco, Invitrogen Carlsbad, CA, United States), 10% v/v fetal calf serum (FCS; Natocor, Córdoba, Argentina), and 100 mg/ml penicillin-streptomycin (Gibco, Invitrogen Carlsbad, CA, United States). Cultures obtained with this procedure showed >98% Iba1 (+) microglia as previously described (Traetta et al., 2021). Microglial cultures from control and VPA animals were challenged with 20 ng/ml of lipopolysaccharide (LPS; *E. Coli* O26:B6 - L3755, Sigma-Aldrich Co.) for 24 h or cortical synaptosomes (ST, synaptic terminals) obtained from naïve adult male Wistar rats (Phillips et al., 2001) for 4 h as previously described (Traetta et al., 2021).

Cells still attached after the first 24 h shaking were subjected to a second 24 h shaking to detach remaining microglia and oligodendrocyte precursors. Then, cells were incubated with 0.625% v/v 5-fluorouracil (Gibco, Invitrogen Carlsbad, CA, United States) for 24 h and washed and incubated in DMEM supplemented with 10% v/v FCS and 100 mg/ml penicillin-streptomycin for an additional 2 DIV. Then, cells were trypsinized (Gibco, Invitrogen Carlsbad, CA, United States) and re-seeded on poly-d-lysine-coated glass coverslips at a density of 2.5 × 10^4^ cells/cm^2^ for treatments and immunofluorescence assays or on plastic dishes for flow cytometry. Astroglial-enriched cultures obtained with this procedure showed >93% GFAP (+) astrocytes, as reported in the literature (Villarreal et al., 2014; Zárate et al., 2019).

For mixed astroglial-microglial cultures, once cells reached confluence, they were tripsinized and re-seeded on poly-d-lysine coated glass coverslips at a density of 2.5 × 10^4^ cells/cm^2^ for treatments and immunofluorescence assays. They were kept in DMEM supplemented with 10% v/v FCS and 100 mg/ml penicillin-streptomycin for an additional 2 DIV. Mixed glial cultures showed 60% GFAP (+) astrocytes, as previously reported (Rosciszewski et al., 2019). Mixed glial cultures (DIV2) from control and VPA animals were also treated for 4 h with cortical synaptosomes (ST, synaptic terminals) obtained from naïve adult male Wistar rats (Chung et al., 2013).

***Cortical neuron-microglia co-cultures:*** cortical neuronal and microglia cultures were performed as described above. Isolated microglia obtained from mixed cultures of control and VPA animals were seeded at a density of 4 × 10^4^ cells/cm^2^ on cortical neurons (DIV4) from control or VPA animals, leading to 4 experimental groups: neurons and microglia from control animals, neurons and microglia from VPA animals, neurons from control animals and microglia from VPA animals, and neurons from VPA animals and microglia from control animals. The neuronal and microglial co-cultures were maintained in a Neurobasal Medium supplemented with 2% v/v B27 and 0.5 mM glutamine at 37°C and 5% CO_2_ for 5 days. In addition, microglia and neurons from control and VPA animals were seeded on coverslips as basal conditions as described for primary cultures, but this time, they were kept in culture for the same time and under the same conditions as co-cultures.

### Immunofluorescence

***Tissue sections:*** control and VPA animals were fixed at PND3 and PND35 as previously detailed (Traetta et al., 2021). At PND3, animals were decapitated, and brains were fixed by immersion in 4% w/v paraformaldehyde in 0.1 M phosphate buffer at 4°C for 24 h. At PND35, animals were anesthetized (125 mg/kg ketamine hydrochloride and 10 mg/kg xylazine, i.p.), transcardially perfused with heparinized saline solution and fixed with 4% w/v paraformaldehyde in 0.1 M phosphate buffer. Brains (PND35) were post-fixed in the same fixative solution at 4°C for 3 h and brains from both PND3 and PND35 animals were equilibrated in 30% w/v sucrose in 0.1 M phosphate buffer before being stored at −70°C. Immunofluorescence technique was performed on 35 μm thick coronal slices of the prefrontal cortex (PFC) corresponding to plates 6–9 (from bregma 4.20 to 2.70) of the atlas of Paxinos and Watson (Paxinos and Watson, 1986) as previously described (Codagnone et al., 2015). Slices were permeabilized (0.5% v/v Triton X-100) and blocked (3% v/v normal horse serum). They were incubated with primary antibodies at 4°C for 48 h and with fluorescent secondary antibodies at room temperature for 1 h. The following primary antibodies were used: anti-synaptophysin (SYN; Millipore Cat# MAB329, RRID: AB_94,786) (1:3,000), anti-vGLUT-1 (Millipore Cat# AB5905, RRID: AB_2,301,751) (1:5,000), anti-glial fibrillary acidic protein (GFAP; Dako Cat# Z0334, RRID: AB_10,013,382) (1:2000), and anti-Iba1 (Wako Cat# 019–19,741, RRID: AB_839,504) (1:4,000) and were followed by fluorescent secondary antibodies (Jackson ImmunoResearch Laboratories, Inc.). Slices were mounted using Mowiol 4–88 (Sigma-Aldrich Co.). Each immunofluorescence assay consisted of 3 PFC serial sections of 4-6 control or VPA animals from 4 different saline and 4 VPA-injected dams.

***Cell culture:*** cortical neurons (DIV7), microglia (DIV7), astrocytes (DIV2), mixed glial cultures (DIV2), and neuron-microglia co-cultures (DIV9) were fixed and processed for immunofluorescence as previously described (Reinés et al., 2012; Podestá et al., 2014). Time points for fixation correspond to fully differentiated cells in culture. Briefly, cells were fixed in 4% w/v paraformaldehyde/4% w/v sucrose in phosphate-buffered saline for 20 min. After permeabilization (0.1% v/v Triton X-100) and blockade (5% v/v normal horse serum), cells were subsequently incubated with primary antibodies overnight at 4°C and with fluorescent secondary antibodies for 1 h at room temperature. The following primary antibodies were used: anti-SYN (1:5,000), anti-microtubule-associated protein 2 (MAP-2; Sigma-Aldrich Cat# M4403, RRID: AB_477,193) (1:500), anti-PSD-95 (Thermo Fisher Scientific Cat# MA1-045, RRID: AB_325,399) (1:200), anti-GAD-67 (Millipore Cat# MAB5406, RRID: AB_2,278,725) (1:1,000), anti-GFAP (1:2000), and anti-Iba1 (1:1,500), followed by fluorochrome-conjugated secondary antibodies (Jackson ImmunoResearch Laboratories, Inc.). 4′,6-Diamidino-2-phenylindole dihydrochloride (DAPI; Sigma-Aldrich Co.) (0.5 μg/ml) was used for the nucleus staining. Coverslips were mounted using Mowiol 4–88 (Sigma-Aldrich Co.). Assays were repeated 2–3 times, employing independent cultures.

### Imaging and Analysis

Immunofluorescence images were captured by an epifluorescence Olympus IX81 microscope equipped with a CCD model DP71 digital camera (Olympus). Image analysis was performed with ImageJ (NIH) software. Quantification parameters were kept constant between groups for analysis, and no blinding method was applied. Final figures were created with Photoshop CS6. In the case of bright and contrast adjustments, they were equally applied to all groups.

***Tissue sections*:** two adjacent non-overlapping images were taken from each hemisphere to sample the medial PFC of animals at PND3 and PND35 according to the atlas of Paxinos and Watson (Paxinos and Watson, 1986). SYN, vGLUT-1, and GFAP immunolabeling were measured as positive immunoreactive areas relative to the total area of the field of view (relative immunoreactive area). To assess the immunoreactive area, we transformed images into grayscale and set a threshold capable of differentiating positive immunoreactive structures from the background. This threshold was kept constant between experimental groups, and the areas of positive structures were those that exceeded the threshold (Codagnone et al., 2015). Iba1 immunolabeling analysis included quantifying the relative immunoreactive area and assesing microglia morphology by classifying Iba1 (+), cells into ramified and unramified categories according to developmental stage (Ueno et al., 2013). At PND3, ramified microglia presented two or more branches, which in turn had more small branches, and unramified microglia were ameboid or showed one or two short, simple branches. At PND35, ramified microglia were considered when a cell presented small soma and more than two long and thin processes, whereas unramified microglia showed larger soma and few short and thick processes.

***Neuronal parameters in culture*:** the immunoreactive pattern generated by synaptic proteins (SYN, PSD-95, and GAD-67) was analyzed as previously detailed (Podestá et al., 2014; Traetta et al., 2021). Briefly, synaptic puncta were captured, setting a single threshold for all experimental groups to distinguish individual puncta from the background and to minimize the probability of including merged structures in the quantification and quantified defining a particle size according to each marker (0.15–0.6 µm^2^ for SYN and PSD-95 and 0.4–1.2 µm^2^ for GAD-67). The quantitative analysis included the total puncta number per neuron indicative of synapse number, the average area occupied by a single punctum (individual puncta area), and the total area occupied by all puncta. Characterization of dendritic arborization consisted of assessing the dendritic tree area by subtracting the area of the soma from the total MAP-2 area, the length of the longest primary dendrite, the number of primary dendrites, and the number of secondary dendrites. Synapses relative to the dendritic tree were quantified as the SYN puncta number relative to a standardized dendrite length (30 µm).

***Microglial morphological studies in culture:*** since microglia adopted different forms according to culture conditions, different parameters were assessed to study their morphology. Microglia [Iba1 (+) cells] morphology in culture and its changes based on the response to different stimuli (LPS or ST) were assessed by measuring the mean area and circularity per field (445 × 335 µm^2^) using ImageJ (NIH) software as previously described (Traetta et al., 2021). These parameters reflect changes in either the extent of individual cell reactivity or the proportion of cells with a reactive profile. In the case of neuron-microglia co-cultures, microglia [Iba1 (+) cells] were classified into four different morphological types according to the number of branches and the size of the soma: cells with long and fine branches as type I, ameboid and large cells as type II, small with one or two very small branches as type III, and small, rounded and unbranched cells as type IV. In the case of mixed cortical glial cultures, microglia [Iba1 (+) cells] were classified into ramified (abundant long or short branches) or unramified (ameboid or bipolar) categories. Results of co-cultures and mixed glial cultures are expressed as mean values of the percentage of each morphology per field. Each field analyzed for neuron-microglia co-cultures corresponded to 445 × 335 µm^2^ and each field for mixed glial cultures to 890 × 670 μm^2^.

***Astrocyte studies in culture:*** cortical astrocytes in astroglial-enriched and mixed cultures were classified according to their morphology as previously described (Acaz-Fonseca et al., 2019; Rosciszewski et al., 2019). Astrocytes [GFAP (+) cells] were considered polygonal if they lacked pronounced cytoplasmic protrusions, bipolar if they presented an elongated cell body or one long thin projection, or stellate if they had a smaller cell body and three or more long processes. Results are expressed as mean values of the percentage of each morphology per field (890 × 670 µm^2^).

### Transmission Electron Microscopy and Analysis

At PND35, control and VPA animals were fixed for electron microscopy as previously described (Traetta et al., 2021; Reinés et al., 2012). Briefly, they were anesthetized as detailed above and transcardially fixed with 4% w/v paraformaldehyde and 2.5% v/v glutaraldehyde in 0.1 M phosphate buffer. Sections were post-fixed in 0.05% w/v osmium tetroxide and dehydrated and embedded in Durcupan. The number of animals employed is in accordance with the literature (Kim et al., 2013; Xu et al., 2020). Sections of the medial PFC from 4 control and 4 VPA animals were contrasted with uranyl acetate and lead citrate as previously detailed (Traetta et al., 2021; Reinés et al., 2012) and photographed with a Zeiss 109 electron microscope equipped with a Gatan W10000 digital camera. Synapse number was assessed throughout 300 µm^2^ of the medial PFC using the ImageJ (NIH) cell counter plugin and expressed as synapse number per µm^2^. An average of 70 and 105 synapses per animal were found in control animals and the VPA group, respectively. The counting criterion was a pre-synapse with visible pre-synaptic vesicles and a prominent post-synaptic density indicative of an excitatory synapse morphology (Stewart et al., 2014).

### Flow Cytometry and Analysis

Cultured cortical microglial cells (DIV7) and astrocytes (DIV2) isolated from control and VPA animals were harvested, fixed, and run on a Partec PAS III flow cytometer (Partec, GmbH, Münster, Germany), as previously described (Zárate et al., 2019; Traetta et al., 2021). Data analysis was performed with WinMDI 98 software. Based on the control group, we selected a representative region on a dot plot and then we used that same region to analyze the VPA group. Forward Scatter (FSC) and Side Scatter (SSC) mean values were used to determine relative cell size and internal complexity (granularity), respectively. Moreover, microglia and astrocytes from two independent cultures were evaluated separately. The high number of cells required per condition limited the repetitions of the experiments performed. A representative experiment of each cell type is shown.

### Reverse Transcription Polymerase Chain Reaction Assays

RT-PCR assays were performed as previously described (Rosciszewski et al., 2018). RNA was isolated using the Quick-RNA™ Miniprep Kit (ZYMO Research, United States). The cDNA was generated using the MMLV-RT Kit (Genbiotech, Argentina) with Oligo(dT)18 Primers (Genbiotech, Argentina). PCR assays were performed using specific primers: TBP (Fwd: ACCGTGAAT CTTGGCTGTAA; Rev: CCG​TGG​CTC​TCT​TAT​TCT​CA; amplification product: 114 bp); IL1β: (Fwd: TCC​ATG​AGC​TTT​GTA​CAA​GG; Rev: GGT​GCT​GAT​GTA​CCA​GTT​GG; amplification product: 246 pb); TNFα: (Fwd: GTA​GCC​CAC​GTC​GTA​GCA​AA; Rev: AAA​TGG​CAA​ATC​GGC​TGA​CG; amplification product: 217 bp); IL6: (Fwd: CTCTCCGCAAGAGACTT CAG; Rev: TCT​GAC​AGT​GCA​TCA​TCG​CT; amplification product: 245 bp); Dicer: (Fwd: TTA​ACC​TTT​TGG​TGT​TTG​ATG​AGT​GT; Rev: GGA​CAT​GAT​GGA​CAA​TTT​TCA​CA; amplification product: 94 bp); Drosha: (Fwd: CAA​AGG​CAA​GAC​GCA​CAG; Rev: CAT​AGG​ACG​ACA​CGG​CTT​G; amplification product: 79 bp). TBP, IL1β, TNFα, IL6, and Drosha were amplified by 30 cycles and the annealing temperature was 60°C, while Dicer was amplified by 30 cycles and the annealing temperature was 62°C. PCR products were run in a 1.5% agarose gel and imaged using a Gel Doc EZ imager (Bio-Rad, United States). Each experiment included negative controls, in which MMLV-RT Kit reactions were performed in the absence of reverse transcriptase. Detailed RT and PCR protocols are available from authors upon request. Results shown correspond to 4 independent microglia or astroglial-enriched cultures at DIV7 and DIV2, respectively.

### Statistical Analysis

Statistical analysis was performed using InfoStat software (Facultad de Ciencias Agropecuarias, Universidad Nacional de Córdoba, Argentina). Student’s *t*-test or Mann–Whitney U test was applied when comparing two independent groups. The first was selected if the variables analyzed fulfilled the requirement of normality (Shapiro–Wilk test) and homogeneity of variances (F-test). In the case of comparing more than two groups, the Kruskal–Wallis test was performed. The analyses and levels of significance between groups are detailed in each figure legend. Values for T, W, H, n, degrees of freedom (df), and exact *p* values are shown in Supplementary Tables S1, S2. Statistical significance was set at *p* < 0.05.

## Results

### Cultured Cortical Neurons Isolated From Valproic Acid Rats in the Absence of Glia Mimic the Synaptic Pattern Observed *In Vivo* in the Prefrontal Cortex

**FIGURE 1:**
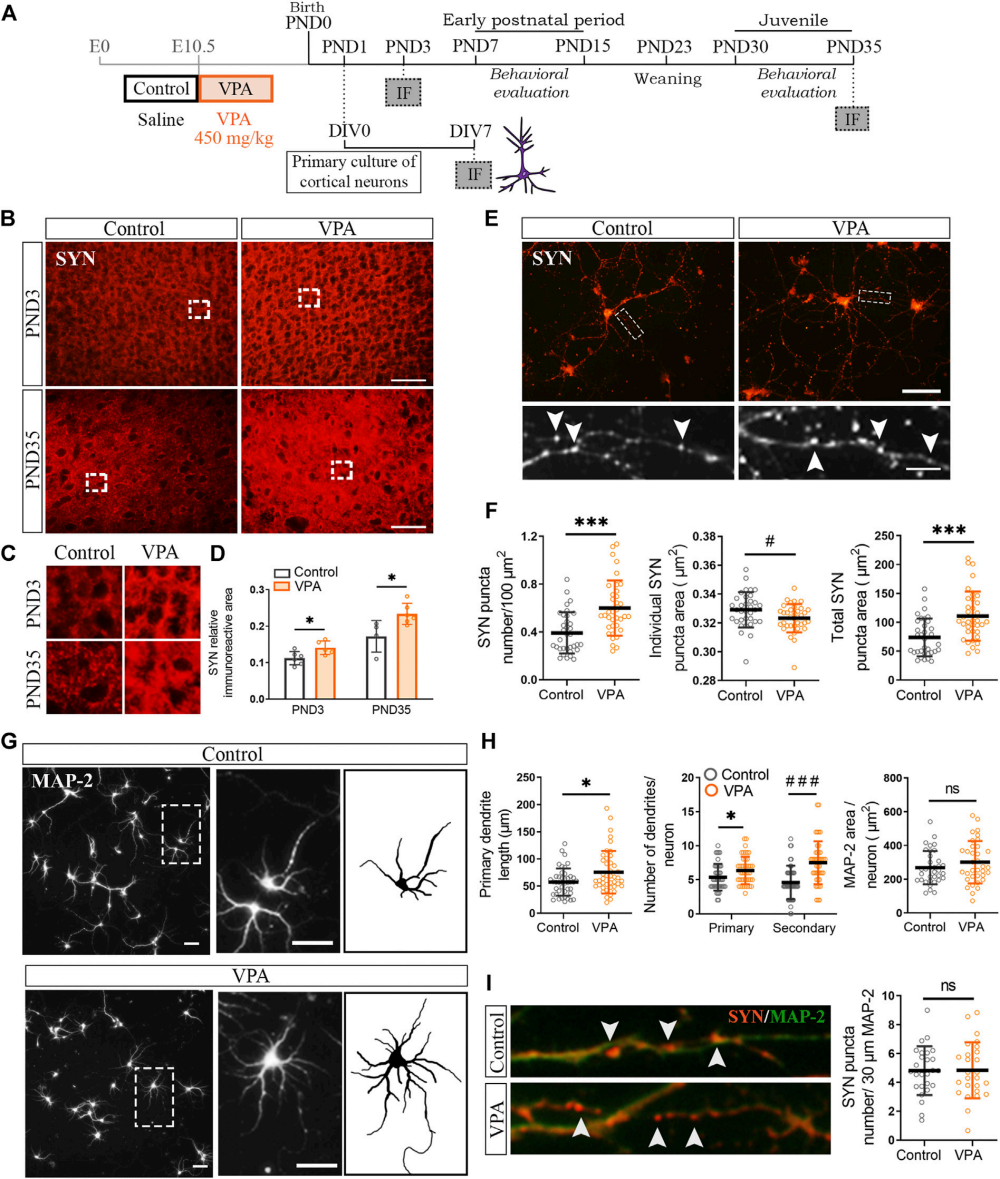
Synaptic changes in the prefrontal cortex (PFC) of VPA rats were present at PND3 and were mimicked *in vitro*, in the absence of glia, by cortical neurons isolated from VPA rats. **(A)** Experimental groups were defined by prenatal VPA injection and pups were behaviorally evaluated. Primary cortical neuronal cultures were prepared from postnatal day (PND) 1 control and VPA pups, kept in culture until day *in vitro* (DIV) 7 and processed for immunofluorescence (IF). At PND3 and PND35, brains from control and VPA animals were processed for IF. **(B)** The PFC from control and VPA animals immunostained for synaptophysin (SYN) at PND3 and PND35. **(C)** Magnifications (×3) of SYN immunostaining revealed a punctate pattern. **(D)** Quantification showed an increase in SYN relative immunoreactive area in the PFC of VPA rats at PND3 and PND35. **(E)** Cortical neurons isolated from control and VPA animals immunostained for SYN. Magnification details SYN immunostaining pattern and arrowheads indicate individual puncta. **(F)** Neurons isolated from VPA animals showed an increase in SYN puncta number, a decrease in individual puncta size, and an increase in total SYN puncta area. **(G)** Cortical neurons isolated from control and VPA animals immunostained for MAP-2; a magnification of an individual neuron from each group and a scheme highlighting its dendritic arborization. **(H)** Quantification revealed longer primary dendrites and an increase in the number of primary and secondary dendrites in neurons from VPA animals. Total area of the dendritic tree did not differ between groups. **(I)** Photomicrographs display dendrites co-immunostained for SYN and MAP-2. Quantification revealed no statistically significant differences in SYN puncta number relative to the dendritic length between groups. Results are expressed as mean values (±SD). **(D)** Control, *n* = 4–6 animals; VPA, *n* = 5–6 animals. **(F,H)** Control, *n* = 34–44 neurons; VPA, *n* = 34–44 neurons from 2 independent cultures. **(I)** Control, *n* = 26 neurons; VPA, *n* = 26 neurons from 2 independent cultures). ns: non-significant, *p < 0.05, ***p < 0.001 between groups by Student’s *t*-test; #*p* < 0.05, ###*p* < 0.001 between groups by Mann–Whitney *U* test. Scale bars: 50 µm **(B,E,G)**; 10 µm (inset **(E)**); the length of the photomicrographs in **I** corresponds to 30 µm.

To address the temporal association of synapse and glial changes in the PFC of VPA rats, we first evaluated SYN immunostaining soon after birth in the neonatal period (PND3) and later during the juvenile postnatal period (PND35) (Figure 1A). Postnatal time points correspond to the postnatal day before the maturation deficits were evident in VPA animals [poor performance in the negative geotaxis test and delayed eye-opening (Supplementary Figures S1A–C)] and the juvenile period, once the social impairment was established in the VPA model (Supplementary Figures S1A,D). The PFC of VPA rats showed increased SYN immunostaining at PND3 and PND35 (Figure 1B), as confirmed by quantification (Figure 1D). SYN immunolabeling showed the typical synaptic pattern that corresponds to protein clusters (Figure 1C). These results indicate that cortical synaptic changes are already evident early during the neonatal period.

Since synaptic alterations observed *in vivo* can be due to either primary neuronal alterations and/or microglial/astroglial altered physiology, we evaluated synapse formation in the absence of glial cells. For that purpose, we cultured cortical neurons isolated from control and VPA animals at PND1 (Figure 1A). Figure 1E shows cortical neurons in culture immunostained for SYN. They display the typical punctate SYN immunostaining that corresponds to synaptic clusters. Primary cortical neurons isolated from VPA rats formed in culture (DIV7) a greater number of synapses than that in controls, as revealed by the increase in SYN puncta number and total SYN puncta area (Figure 1F). Cortical neurons isolated from VPA animals also showed a reduction in individual SYN puncta size indicative of smaller clusters of synaptic vesicles (Figure 1F). As synapse formation and dendritic arborization are tightly associated (Rollenhagen et al., 2013), we studied the dendritic tree of cortical neurons by MAP-2 immunostaining (Figure 1G). Cortical neurons isolated from VPA animals exhibited a more complex dendritic arbor characterized by longer primary dendrites and a greater number of primary and secondary dendrites (Figure 1H). However, these rendered no differences in dendritic tree area that might indicate dendrites of smaller caliber (Figure 1H). Figure 1I shows neurons co-labeled with SYN/MAP-2; quantification of SYN puncta number relative to dendritic length revealed the absence of statistically significant differences between groups. These results imply that *in vitro* and in the absence of glia, postnatal cortical neurons isolated from VPA rats mimic the *in vivo* increase in synapse number and that the higher complexity of dendritic arborization could account for such synapse increment.

### The Greater Number of Excitatory Synapses Characterizes the Cortical Synaptic Profile of Valproic Acid Animals *In Vivo* and *In Vitro*

**FIGURE 2:**
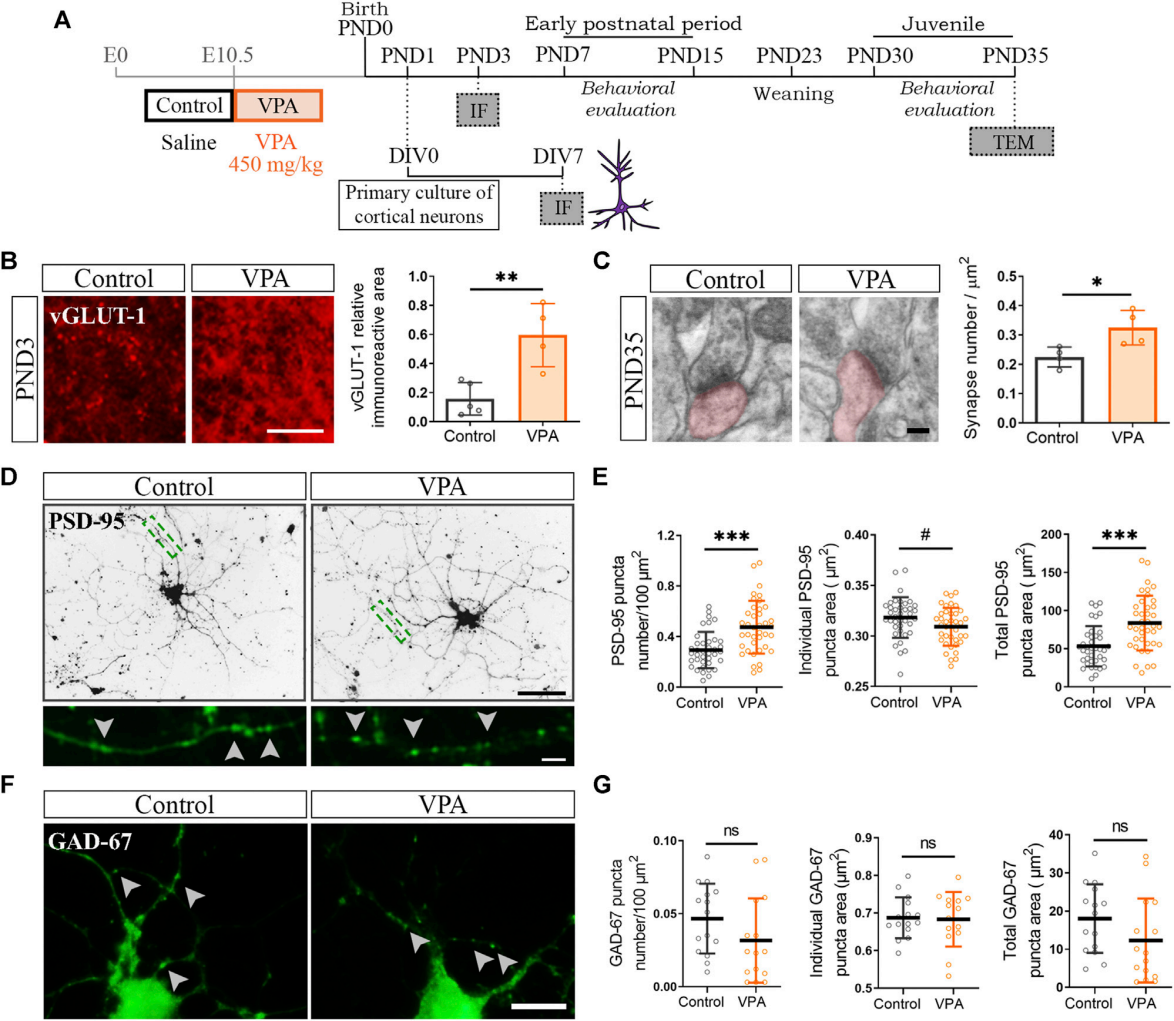
Increased number of excitatory synapses in the prefrontal cortex (PFC) of VPA animals and in cultured cortical neurons isolated from VPA rats. **(A)** Once the VPA model was established, primary cortical neuronal cultures were prepared from PND1 control and VPA pups and processed at day *in vitro* (DIV) 7 for immunofluorescence (IF). The model was validated behaviorally. Brains from control and VPA animals were processed at PND3 for IF and at PND35 for transmission electron microscopy (TEM) analysis. **(B)** At PND3, the PFC of VPA animals displayed an increase in vesicular glutamate transporter 1 (vGLUT-1) relative immunoreactive area. **(C)** At PND35, the PFC of VPA animals exhibited a greater number of excitatory synapses (asymmetric) by TEM. **(D)** Cultured cortical neurons isolated from control and VPA animals immunostained for PSD-95. Magnification depicts a punctate immunostaining pattern. **(E)** Quantification showed an increase in PSD-95 puncta number, a reduced individual puncta size and an increase in total puncta area in neurons from VPA animals. **(F)** Cortical neurons isolated from control and VPA animals immunostained for GAD-67. Arrowheads indicate individual puncta. **(G)** No statistically significant differences were found between groups in any parameter quantified. Results are expressed as mean values (±SD). **(B,C)** Control, *n* = 4–5 animals; VPA, *n* = 4 animals. **(E)** Control, *n* = 38 neurons; VPA, *n* = 39 neurons from 2 independent cultures. **(G)** Control, *n* = 15 neurons; VPA, *n* = 15 neurons from 2 independent cultures. ns: non-significant, *p < 0.05, **p < 0.01, and ***p < 0.001 between groups by Student’s *t*-test and #*p* < 0.05 between groups by Mann–Whitney *U* Test (for individual GAD-67 puncta area). Scale bars: 50 µm **(D)**; 25 µm **(B)**; 10 µm **(F)**; 5 µm (inset **(D)**); 100 nm **(C)**.

To determine the contribution of glutamatergic synapses to the increase in the number of synapses in the PFC of VPA animals, at PND3 and PND35, we evaluated the immunostaining for vesicular glutamate transporter (vGLUT-1) and electron microscopy, respectively (Figures 2A–C). The PFC of VPA rats showed increased vGLUT-1 immunolabeling at PND3 confirmed by quantification of the relative immunoreactive area (Figure 2B). At PND35, quantification of asymmetric synapses revealed a greater number of excitatory synapses in the PFC of VPA rats than that in the controls (Figure 2C). To study excitatory and inhibitory synapses in cortical neurons isolated from control and VPA animals cultured in the absence of glia, cells were immunostained for PSD-95 and GAD-67, respectively (Figures 2A,D,F). Both synaptic markers displayed the characteristic punctate immunostaining (Figures 2D,F, higher magnification). Cortical neurons from VPA animals formed a higher number of excitatory post-synapses in culture, as revealed by the increase in PSD-95 puncta number and total PSD-95 puncta area (Figures 2D,E); post-synapses were smaller as indicated by the reduction in individual PSD-95 puncta area (Figure 2E). These results parallel the alterations in SYN puncta (Figures 1E,F). Regarding GABAergic synapses, no statistically significant differences were found between neurons from control and VPA animals in either GAD-67 puncta number, total area, or individual GAD-67 puncta area (Figures 2F,G). These results show that the PFC of VPA rats exhibits an increase in excitatory synapses in both the neonatal and juvenile periods and that this synaptic pattern is reproduced *in vitro* in the absence of glial cells.

### Cortical Microglia Isolated From Valproic Acid Animals Once Synaptic Changes Are Already Established Show *In Vitro* Long-Lasting Changes

**FIGURE 3:**
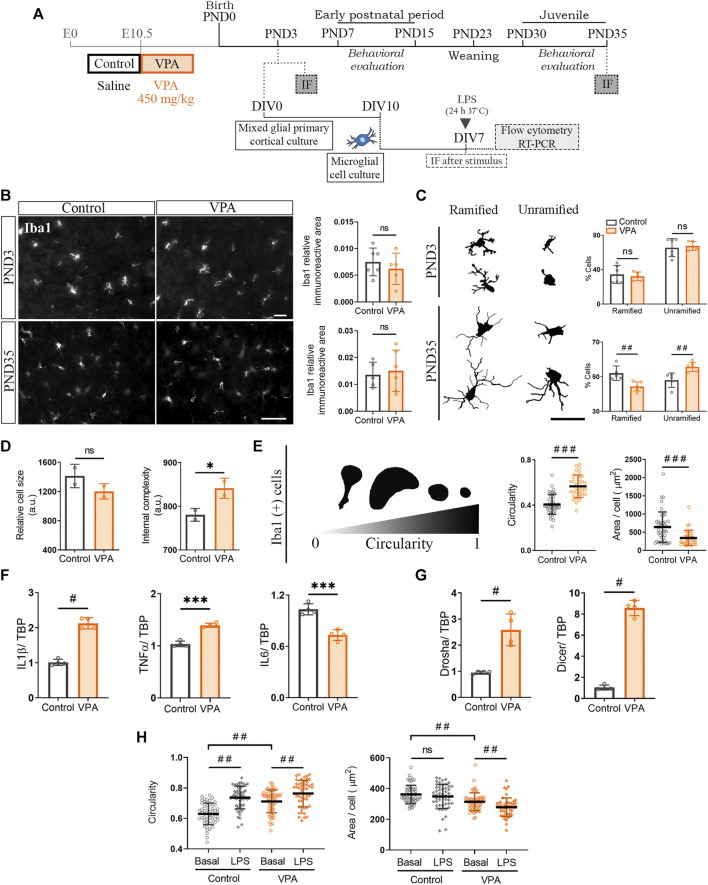
Cultured cortical microglia isolated from VPA animals showed a pro-inflammatory profile which was absent *in vivo* at PND3 but present at PND35. **(A)** Primary mixed cortical glial cultures were prepared from postnatal day (PND) 3 control and VPA animals. At day *in vitro* (DIV) 10, microglia were detached, re-seeded, and grown for 7 days before analysis. Siblings were behaviorally evaluated. At PND3 and PND35, brains from control and VPA animals were processed for immunofluorescence (IF). **(B)** PFC of control and VPA animals immunostained for Iba1. At PND3 and PND35, no statistically significant differences were found in Iba1 relative immunoreactive area. **(C)** Schematic representation of microglial morphology at PND3 and PND35. At PND35 but not at PND3, the PFC of VPA rats evidenced an increase in the proportion of unramified Iba1 (+) cells concomitantly with a decrease of ramified cells. **(D)** Cultured microglia isolated from VPA animals showed preserved size but increased internal complexity when analyzed by flow cytometry. **(E)** Representation of microglia morphology in culture immunostained for Iba1 (DIV7) and its associated circularity. Microglia isolated from VPA animals displayed an increase in circularity and a smaller cell area. **(F)** Microglia isolated from VPA animals showed increased expression of IL1β and TNFα but lower IL6 RNA levels. **(G)** The expression of Drosha and Dicer was increased in microglia isolated from VPA rats. **(H)** After a 24 h LPS stimulus, microglia from both control and VPA animals showed greater circularity, but only microglia from VPA animals showed a smaller size compared with their basal condition. Results are expressed as mean values (±SD). **(B,C)** Control, *n* = 5–6 animals; VPA, *n* = 5–6 animals. **(D)** A representative experiment run by duplicate. **(E)** Control, *n* = 42 photomicrographs; VPA, *n* = 35 photomicrographs from 3 independent cultures; **(F,G)** 4 independent cultures; **(H)** *n* = 50–57 photomicrographs per group from 2 independent cultures. **(B–G)** ns: non-significant (for B,D); *p < 0.05, and ***p < 0.001 between groups by Student’s *t*-test (for C); #*p* < 0.05, ##*p* < 0.01, and ###*p* < 0.001 between groups by Mann–Whitney *U* test; **(H)** ##*p* < 0.01 between groups by Kruskal–Wallis test. Scale bar: 50 µm.

Keeping in mind that synaptic changes are already evident in the neonatal period and persist into the juvenile stage in the PFC of VPA animals, we aimed to analyze microglial morphology early at PND3 and later at PND35 (Figure 3A). Initial quantification of the Iba1 immunostained area showed no statistically significant differences at either PND3 or PND35 between control and VPA animals (Figure 3B). Further meticulous morphology assessment of Iba1 (+) cells revealed a similar proportion of ramified and unramified microglia in control and VPA animals at PND3 (Figure 3C). However, as development proceeds, at PND35, the PFC of VPA rats showed a smaller percentage of ramified microglia and a higher proportion of unramified cells than those of control animals (Figure 3C). These results suggest that microglia show a normal profile at PND3 but turns into reactive microglia at PND35 *in vivo*.

To study microglial cells in the absence of neurons, we prepared primary microglial cultures from control and VPA rats at PND3 (Figure 3A). This time point is appropriate for culturing glial cells (McCarthy and de Vellis, 1980; Tamashiro et al., 2012); in this case, it coincides with the presence of early synaptic changes in the PFC of VPA animals (Figure 1B). Under basal conditions, cortical microglia isolated from VPA animals showed a tendency to be smaller and to have increased internal complexity (Figure 3D) consistent with the decreased cell area and increased circularity found by morphological evaluation of Iba1 (+) cells (Figure 3E). A greater internal complexity, greater circularity, and reduced cell area are characteristic parameters of reactive microglia (Ritzel et al., 2015; Lively and Schlichter, 2018). This reactive microglial morphology matched the pro-inflammatory cytokine profile found in microglia isolated from VPA animals (Figure 3F) (Lively and Schlichter, 2018; Hoogland et al., 2015). Since miRNA processing enzymes are involved in regulating inflammatory responses in microglia (Varol et al., 2017), we studied the expressions of Drosha and Dicer in microglia isolated from control and VPA rats. The expressions of Drosha and Dicer were increased in microglia isolated from VPA animals (Figure 3G). Since the absence of Dicer exacerbates the microglia pro-inflammatory response (Varol et al., 2017), these results may suggest that the microglial cytokine expression profile is regulated by both transcriptional and translational control mechanisms resulting in a mild pro-inflammatory profile. We then evaluated microglial response when exposed to the pro-inflammatory LPS stimulus. Cortical microglia from both control and VPA animals reacted by increasing cell circularity (Figure 3H). This result indicates that even when microglia from VPA animals show a reactive morphological profile under basal conditions, these cells are able to further respond to a pro-inflammatory stimulus and the experimental assay can evidence further increments in microglia reactivity.

Altogether, our results indicate that microglial cells isolated from VPA animals show long-lasting changes when fully differentiated in culture, characterized by a basal reactive profile but still with a preserved response to classical pro-inflammatory LPS. These results suggest that even though microglial alterations *in vivo* seem absent at PND3 and become statistically representative at PND35, isolated microglia at PND3 already show an altered, mild reactive profile when maintained in culture *in vitro*, which do support early microglia modifications at PND3.

### Adaptive Response of Cortical Microglia to Neuronal Milieu in Valproic Acid Rats: Normal Morphological Profile, Preserved Neurite Outgrowth Stimulation, and Resistance to Phagocytic Stimuli

**FIGURE 4:**
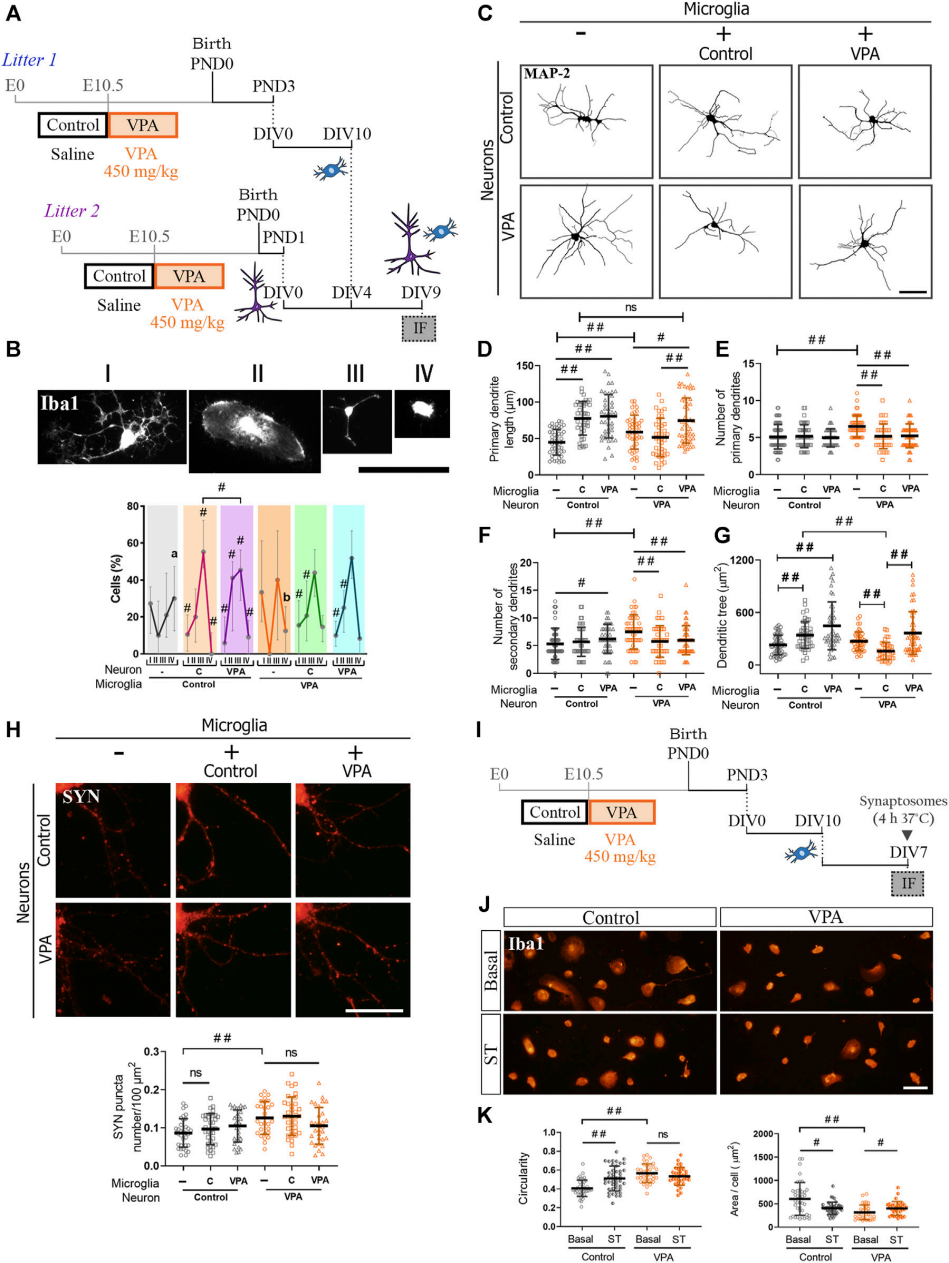
Microglia from VPA animals showed resistance to phagocytic stimuli and, in the presence of neurons isolated from VPA rats, acquired a normal morphological profile and promoted neurite outgrowth. **(A)** Co-cultures were obtained by seeding cortical microglia on cortical neurons (DIV4). After 5 days in co-culture conditions, cells were processed for immunofluorescence (IF). **(B)** Representation of microglial morphology in co-cultures immunostained for Iba1 and its categorization (I–V). In the presence of neurons, microglia isolated from VPA rats showed a similar morphological profile regardless of neuronal substrate, which was similar to microglia from control rats in the presence of neurons from control animals. Microglia isolated from control rats evidenced a different profile when exposed to neurons from VPA animals. **(C)** Dendritic arbor of cortical neurons isolated from control and VPA animals in co-culture conditions and immunostained for MAP-2. **(D)** Except for neurons isolated from VPA animals exposed to microglia from control animals, an increase in the length of primary dendrites was observed in the other conditions. Microglia isolated from control or VPA animals reduced the number of **(E)** primary and **(F)** secondary dendrites of neurons isolated from VPA animals. **(G)** An increase or a trend toward an increase in dendritic tree area was concomitant to the dendritic outgrowth effect. Microglia isolated from control animals reduced the dendritic tree area of neurons from VPA rats. **(H)** Cortical neurons isolated from control and VPA animals immunostained for SYN in the absence and presence of microglia from control and VPA animals. Quantification revealed no statistically significant differences in SYN puncta number in the presence of microglia. **(I)** Primary microglia cultures were exposed to synaptic terminals (ST). **(J)** Microglial culture from control and VPA animals immunolabeled for Iba1 under basal conditions and after exposure to ST. **(K)** Microglia isolated from VPA animals showed greater circularity and a smaller cell area than those in the control group under basal conditions. After exposure to ST, microglia from control animals responded with an increase in circularity and a reduction in the cell area. Microglia from VPA animals responded with a slight increase in cell area. Results are expressed as **(B)** median with interquartile range (*n* = 24–27 photomicrographs per group from 2 independent co-cultures); **(D-H,K)** mean values (±SD): **(D–G)** 39–68 neurons per group from 2 independent co-cultures; **(H)** 29–35 neurons per group from 2 independent co-cultures; **(K)** 34–43 photomicrographs per group from 3 independent cultures). ns: non-significant; #*p* < 0.05; ##*p* < 0.01 between groups by Kruskal–Wallis test. In **(B),** # indicates difference from the microglial culture of that same precedence and different letters show differences between microglia experimental groups. Scale bars: 50 µm **(B,C,J)**; 25 µm **(H)**.

Considering that both neurons and microglia isolated from VPA animals mimic juvenile alterations once differentiated individually *in vitro*, but microglial changes are only evident in the PFC of juvenile VPA animals, we aimed to study how neurons and microglia from control and VPA rats interact *in vitro* in reconstituted cultures. Neuron-microglia interaction was studied in reconstituted co-cultures of primary cortical neurons and microglia isolated from control and VPA rats by evaluating microglial morphology and neuronal parameters (Figure 4A). Since microglial reactivity profile is associated with cell functionality (Walker et al., 2014; Fernández-Arjona et al., 2019), we first determined microglial response to different neuronal microenvironments by co-culturing microglia isolated from control and VPA animals with neurons from control or VPA rats and measuring cell morphology in cells immunostained for Iba1. Representative photomicrographs in Figure 4B show Iba1 (+) cell classification into four morphological categories that were used to study their response to neurons isolated from control or VPA rats. As shown in Figure 4B, microglial cells isolated from control rats revealed a quite different morphological profile when exposed to neurons isolated from VPA rats compared to those with neurons from controls (Figure 4B). Interestingly, microglia isolated from VPA animals showed the same profile when exposed to neurons from control and VPA rats. Indeed, this morphological profile was also similar to that shown by microglia from control animals exposed to neurons from control rats. Thus, we conclude that microglia derived from VPA animals are adapted to the molecular cues shown by neurons derived from VPA animals. Microglial cells from VPA animals are adapted to their neuronal substrate, thus behaving as control microglia in the presence of control neurons.

Since microglia promote neurite outgrowth *in vitro* (Zhang and Fedoroff, 1996), we addressed microglial effects on dendritic arborization in neuron-microglia co-cultures (Figure 4A). Figure 4C shows representative photomicrographs of MAP-2 immunostaining in cortical neurons isolated from control and VPA rats and cultured in the absence or presence of microglia isolated from either experimental group. As expected, microglia from control animals promoted neurite outgrowth in neurons from control animals by inducing longer primary dendrites, leading to an increase in dendritic tree area (Figures 4C,D,G). Likewise, microglia isolated from VPA rats induced neurite outgrowth by increasing the length of primary dendrites in neurons isolated either from VPA or control animals (Figures 4C,D). Accordingly, a trend toward an increase or a significant increase in dendritic tree area was recorded in neurons from VPA and control animals, respectively (Figure 4G). Besides, microglia isolated from control or VPA animals decreased the number of primary and secondary dendrites in neurons from VPA animals but not in neurons from control animals (Figures 4E,F). Surprisingly, while microglia from VPA animals promoted dendritic outgrowth on any neuronal substrates, microglia isolated from control animals did not increase dendritic length and even reduced the dendritic tree area of neurons isolated from VPA rats (Figures 4D,G). These results indicate that microglia isolated from VPA animals induce similar dendritic effects on neurons from control or VPA animals and these resemble those induced by microglia from control animals on neurons isolated from control animals. Moreover, microglia from control animals induce distinctive effects on neurons isolated from VPA rats by eliciting opposing changes to those produced by microglia from VPA animals. Thus, microglia from VPA animals seem to be adapted to the neuronal milieu; consequently, they respond as normal microglia even on neurons exhibiting synaptic and dendritic changes.

As microglia also play a role in synapse formation and pruning (Paolicelli et al., 2011; Schafer et al., 2012; Miyamoto et al., 2016), we studied the effect of microglia isolated from control and VPA animals on SYN puncta number in neurons in these co-culture conditions from both experimental groups (Figures 4A,H). As previously shown in Figure 1F, neurons isolated from VPA animals showed an increase in SYN puncta number in the absence of microglia (Figure 4H). In our experimental conditions, microglia from control animals did not modify SYN puncta number in neurons from either control or VPA rats. Similarly, microglia from VPA rats did not affect SYN puncta number irrespective of the neuronal substrate. These results suggest that microglia from VPA animals have no counteracting effect on the increase in synapse number shown in neurons from VPA rats. Thus, we evaluated microglia response under a phagocytic stimulus induced by exposure to synaptic terminals (Figures 4I,J). To assess a general response, isolated cortical microglia from control and VPA animals were exposed to cortical synaptosomes purified from naïve rats and then immunostained for Iba1. Microglia isolated from control animals and exposed to synaptic terminals showed increased circularity and reduced cellular area (Figure 4K), both changes indicative of cell reactivity. Interestingly, microglia isolated from VPA animals did not show a statistically significant morphological response after exposure to synaptic terminals (Figures 4J,K), even when these cells increased their circularity in response to LPS (Figure 3H). These results indicate that cortical microglia isolated from VPA animals cannot alter synapse number when co-cultured with neurons and show resistance to the phagocytic stimulus by synaptosomes. Overall, results suggest that microglia isolated from VPA animals are less sensitive or adapted to their neuronal milieu, adopting a normal morphological profile, promoting neurite outgrowth, and showing a lack of response to phagocytic cues.

### Cortical Astrocytes Isolated From Valproic Acid Rats Are Engaged in a Long-Lasting Pro-Inflammatory Program

**FIGURE 5:**
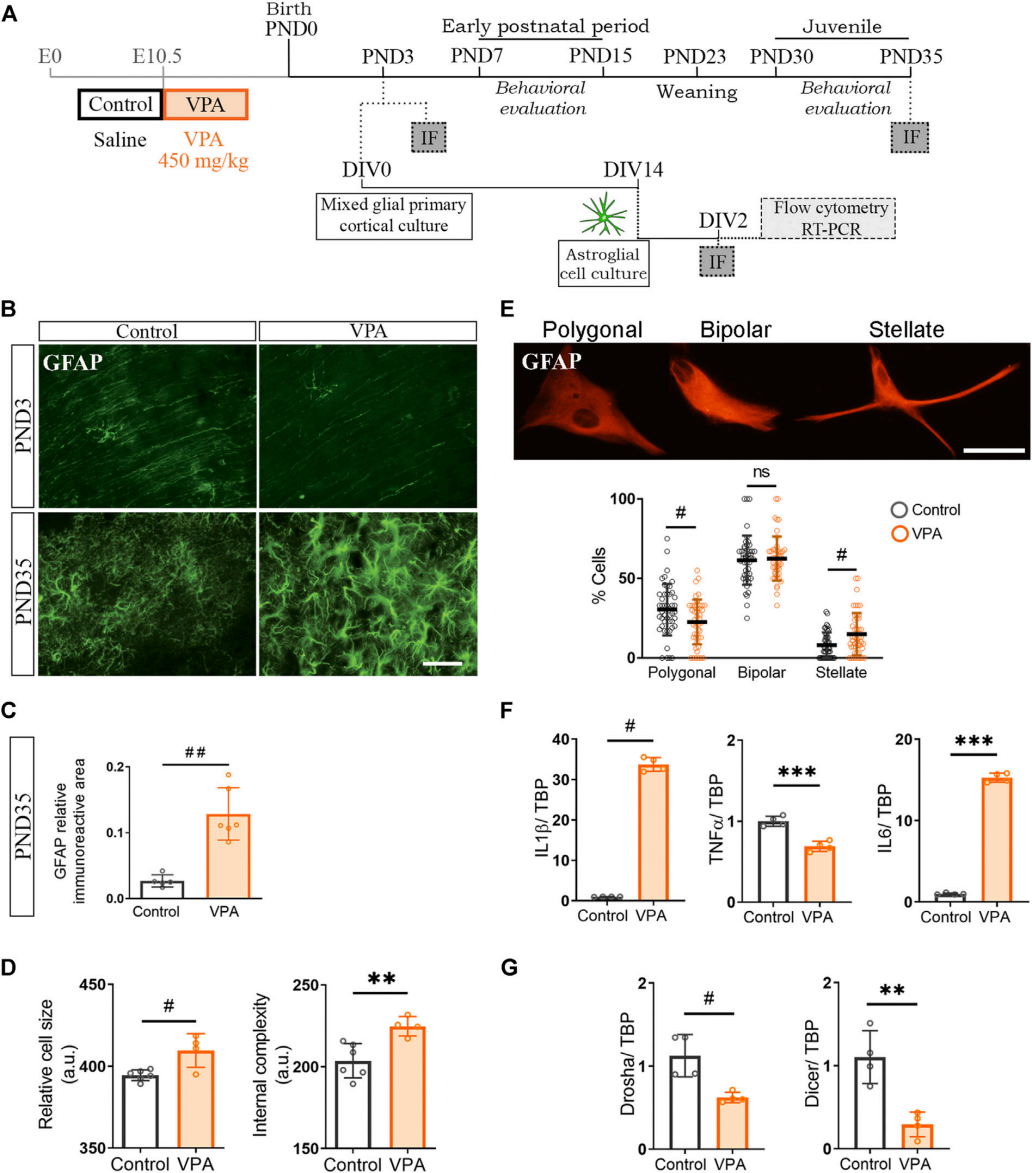
Cultured cortical astroglia isolated from VPA animals showed an inflammatory profile that was revealed *in vivo* at PND35. **(A)** Mixed cortical glial cultures were prepared from postnatal day (PND) 3 control and VPA rats to obtain astroglial-enriched cultures, which were processed [day *in vitro* (DIV) 2] for flow cytometry, RT-PCR, and immunofluorescence. Siblings were behaviorally evaluated. At PND3 and 35, immunofluorescence (IF) was performed on brains from control and VPA animals. **(B)** The PFC of control and VPA rats immunostained for GFAP at PND3 and 35. At PND3, the immunostaining pattern corresponded to radial glia and at PND35 to astrogliosis in VPA animals. **(C)** Quantification at PND35 confirmed an increase in GFAP relative immunoreactive area in the PFC of VPA animals. **(D)** Cortical astrocytes in culture isolated from VPA animals showed an increase in cell size and internal complexity when analyzed by flow cytometry. **(E)** Representation of astrocyte morphology in culture immunostained for GFAP and its corresponding categorization. Astrocytes isolated from VPA animals displayed a reduction in the proportion of polygonal cells with a concomitant increase in stellate astrocytes. **(F)** Astrocytes isolated from VPA animals showed increased expression of IL1β and IL6 but lowered TNFα RNA levels. **(G)** The expression of Drosha and Dicer was diminished in astrocytes isolated from VPA rats. Results are expressed as mean values (±SD). **(C)** Control, *n* = 5 animals; VPA, *n* = 6 animals. **(D)** A representative experiment. **(E)** Control, *n* = 47 photomicrographs; VPA, *n* = 47 photomicrographs from 3 independent cultures. **(F,G)**: 4 independent cultures, **p < 0.01, ***p < 0.001 between groups by Student’s *t*-test and ns: non-significant; #*p* < 0.05; ##*p* < 0.01 between groups by Mann–Whitney *U* test. Scale bar: 50 µm.

Our results show that cortical microglia from VPA animals exhibit a normal morphological profile when exposed to neurons from VPA animals *in vitro*. However, these cells display a reactive morphology in the PFC of VPA rats at PND35 *in vivo*. Since astrocytes are known to modulate the microglial function (Matejuk and Ransohoff, 2020; Liddelow et al., 2020) and, in the PFC of VPA animals, these glial cells exhibit a reactive morphology at PND35 (Codagnone et al., 2015), we hypothesized that astroglia-microglia crosstalk could be responsible for the long-term reactive microgliosis *in vivo* in VPA animals. We first determined the temporal relationship of astroglial morphology with respect to synaptic changes by evaluating GFAP immunostaining in the PFC of control and VPA rats at PND3 and PND35 (Figure 5A). At PND3, the PFC of VPA rats showed a GFAP pattern compatible with immature glia (radial glia) and similar to age-matched control animals (Farhy-Tselnicker and Allen, 2018). At PND35, GFAP immunostaining was increased in the VPA group (Figure 5B) as confirmed by quantification of relative immunoreactive area (Figure 5C), which is indicative of astrogliosis (Hol and Pekny, 2015). These results suggest that cortical astrogliosis is evident after synaptic changes are established in the PFC (PND3). Then, to study astrocytes in the absence of neurons and microglia, we isolated cortical astrocytes from control and VPA animals (PND3) and grew them in culture (Figure 5A). Astrocytes isolated from the VPA group showed increased cell size and internal complexity assessed by flow cytometry (Figure 5D). Morphological evaluation of astrocytes in culture immunostained for GFAP revealed an increased proportion of stellate cells with a concomitant decrease in the proportion of polygonal astrocytes in the VPA group (Figure 5E). Stellate morphology, larger astrocytes, and higher internal complexity are parameters of increased cell reactivity (Rosciszewski et al., 2019; Acaz-Fonseca et al., 2019). In accordance with the reactive morphological profile, cortical astrocytes isolated from VPA rats exhibited a pro-inflammatory cytokine expression profile (Figure 5F). We next evaluated miRNA processing enzymes and found that astrocytes isolated from VPA animals showed a reduced expression of Drosha and Dicer (Figure 5G). This alteration can be associated with a pro-inflammatory program since Dicer deficiency proved to lead to astrogliosis (Sun et al., 2019). Altogether, these results indicate that cortical astrocytes isolated from VPA animals show long-lasting changes that give rise to a reactive profile once fully differentiated *in vitro* and this parallels with astrogliosis observed in the PFC of VPA animals at PND35. Considering that major astrogliogenesis occurs after the neonatal period (Farhy-Tselnicker and Allen, 2018) and that astrocytes are crucial for modulating microglial function (Matejuk and Ransohoff, 2020), long-lasting changes in astrocytes could affect microglia and, thus, contribute to the microglial reactive profile seen in the PFC of VPA rats at PND35.

### Distinctive Cortical Astrocyte-Microglia Crosstalk in Valproic Acid Rats

**FIGURE 6:**
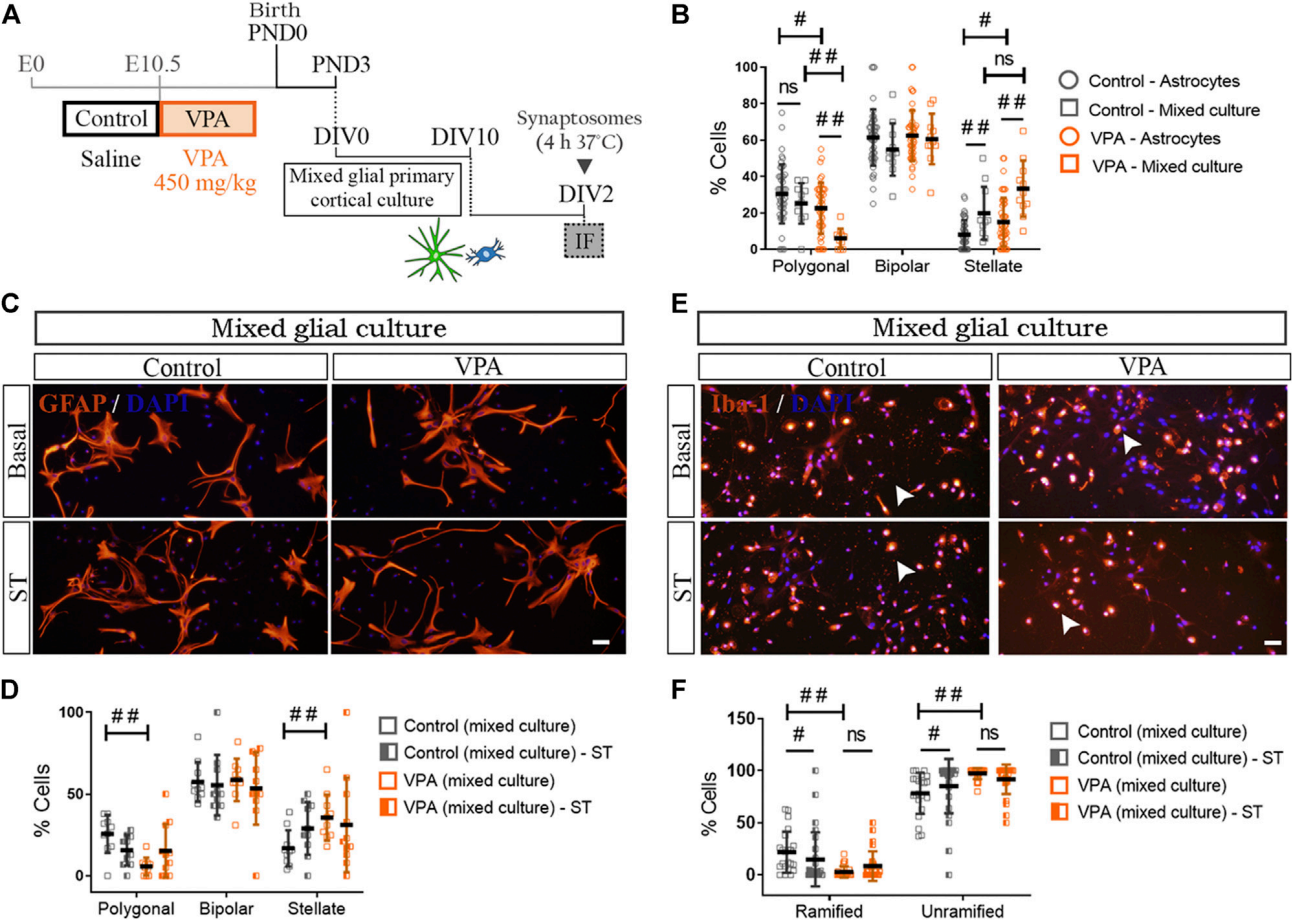
Impact of astrocyte-microglia crosstalk on morphological profiles and responses to synaptic terminals of glial cells isolated from control and VPA animals. **(A)** Mixed cortical glial cultures isolated from control and VPA animals were exposed to synaptosomes (ST) (DIV2). **(B)** In mixed glial cultures from control animals, the presence of microglia increased the proportion of stellate astrocytes compared with astroglial-enriched cultures (astrocytes). In the VPA group, the presence of microglia increased stellate and decreased polygonal astrocytes when compared with astroglial-enriched cultures (astrocytes). **(C)** Mixed glial cultures from control and VPA animals under basal conditions and after exposure to cortical ST immunostained for GFAP and labeled with DAPI. **(D)** In mixed glial cultures, astrocyte morphology is not altered by exposure to ST in either control or VPA group. **(E)** Mixed glial cultures from control and VPA animals immunostained for Iba1 and labeled with DAPI under basal conditions and after exposure to cortical ST. **(F)** While in mixed glial cultures from control animals exposed to ST, microglia responded with a decrease in ramified cells and an increase in unramified cells, in mixed cultures isolated from VPA animals, microglia failed to respond. Results are expressed as mean values (±SD) **(B)**: 11 photomicrographs per group from two independent mixed cultures and 47 photomicrographs per group from three independent astroglial-enriched cultures. **(D,F)** 10–26 photomicrographs per group from 2 independent mixed cultures; ns: non-significant; #*p* < 0.05; ##*p* < 0.01 between groups by Kruskal–Wallis test. Scale bars: 50 µm.

Our previous results suggest that long-lasting changes in cortical astrocytes isolated from VPA animals may affect the astroglial function and consequently astroglia-microglial crosstalk (Matejuk and Ransohoff, 2020). To address astrocyte-microglia bidirectional communication, we studied astrocyte morphology in mixed cortical glial cultures immunostained for GFAP (Figures 6A,B). In mixed glial cultures isolated from control animals, the presence of microglia increased the proportion of stellate cells without modifying the proportion of polygonal astrocytes [astroglial-enriched cultures (astrocytes) vs. mixed cultures] (Figure 6B). It should be noted that, as also shown in Figure 5E, astrocytes isolated from VPA animals exhibited an increased proportion of stellate astrocytes concomitantly with a reduction in the proportion of polygonal cells in astroglial-enriched cultures [astroglial-enriched cultures (astrocytes) vs. mixed cultures] (Figure 6B). In these astrocytes, the presence of microglia further increased the proportion of stellate cells with a concomitant reduction in the proportion of polygonal astrocytes (Figure 6B). Figure 6D shows quantification of astrocyte morphology in cortical mixed cultures from control and VPA animals exposed to synaptic terminals. In the presence of microglia, exposure to synaptic terminals did not promote morphological changes in astrocytes isolated from either control or VPA rats when compared with basal mixed culture conditions (Figures 6C,D). Thus, results indicate that the presence of microglia exacerbates the reactive morphological profile of astrocytes isolated from VPA rats toward an increase in stellate cells and a reduction in polygonal cells, indicative of astrocyte reactivity. Results suggest that cortical astroglia-microglia crosstalk in the VPA model results in greater astrocyte reactivity.

We then evaluated astrocyte-microglia crosstalk by assessing microglia morphology in Iba1 (+) cells from mixed cortical glial cultures (Figures 6E,F). In the presence of astrocytes, microglia from VPA animals showed increased basal reactivity as indicated by a lower proportion of ramified microglia and a higher proportion of unramified cells in comparison with mixed glial cultures from control rats (Figure 6F). It is worth mentioning that a similar microglia profile was found in microglia cultures from VPA animals (Figure 3E). In the presence of astrocytes, exposure to synaptic terminals induced a reduction in ramified cells concomitantly with an increase in the proportion of unramified microglia in the control group (Figure 6F) (Bohatschek et al., 2001). However, such an effect was not observed in microglial cells in mixed cultures from VPA animals (Figure 6F). It should be mentioned that microglia isolated from VPA rats were also insensitive to a phagocytic stimulus (Figure 3F). Results indicate that microglia display a reactive profile in the presence of astrocytes and that astrocyte-microglia crosstalk does not engage microglia in a phagocytic response.

## Discussion

This study provides evidence of long-lasting microglia and astroglia changes induced by prenatal exposure to VPA and suggests microglia-astroglia crosstalk as a key process implicated in neuroinflammation described for ASD. Our findings reveal that cortical microglia isolated from VPA animals are insensitive or adapted to synaptic changes and that microglia-astroglia crosstalk enables microglia pro-inflammatory profile and exacerbates astrogliosis without engaging microglia in response to phagocytic stimuli. In the present work, we used the VPA rat model since it mimics behavioral, anatomical, synaptic, and glial patterns described in ASD (Nicolini and Fahnestock, 2018) and studied synaptic and glial profiles in the PFC, a brain area associated with ASD impairments (Benekareddy et al., 2018; Chini and Hanganu-Opatz, 2021). Herein, we show that in the PFC of VPA animals, synaptic changes are evident in the neonatal period, but there is an absence of microgliosis. *In vitro*, cortical microglia isolated from VPA animals, when synaptic changes are already established, show a long-lasting but mild pro-inflammatory profile and an intrinsic resistance to phagocytic stimuli when cultured in the absence of neurons. Cortical microglia show a biphasic response, with initial unresponsiveness *in vivo* and *in vitro* to the synaptic alterations presented by neurons from VPA animals and a late microgliosis in juvenile animals. Specifically, in the presence of neurons, cortical microglia display a normal morphological pattern and promote dendritic outgrowth. In line with their adaptive response, microglia show an increase in miRNA processing enzymes that may participate in anti-inflammatory processes. However, *in vivo*, juvenile VPA animals show microgliosis in the PFC. Interestingly, the microgliosis at the juvenile period of VPA animals is concomitant with astrogliosis. Astrocytes isolated from VPA animals show a broader long-lasting pro-inflammatory profile, including an increased expression of pro-inflammatory cytokines and a decrease in miRNA processing enzymes. More importantly, microglia-astroglia crosstalk enables microglia pro-inflammatory profile and exacerbates astrogliosis without engaging glial cells in response to phagocytic stimuli.

Regarding the synaptic changes in the PFC, juvenile VPA animals evidence a greater number of excitatory synapses. This is in accordance with previous reports showing increased glutamatergic synaptic proteins (Kim et al., 2013) and hyper-connectivity and hyper-plasticity (Rinaldi et al., 2008) in this brain region of juvenile VPA animals. Interestingly, PFC activation impairs social behavior (Benekareddy et al., 2018). Remarkably, this alteration is revealed early during the postnatal period since SYN and vGLUT-1 immunolabeling increase at PND3. Particularly, an increase in vGLUT-1 expression could also suggest a different maturation synaptic stage in this brain region of VPA animals (Minelli et al., 2003; Farhy-Tselnicker and Allen, 2018). Similarly, electrophysiological alterations in the PFC of VPA animals have been reported early in development (Walcott et al., 2011). This synapse pattern seen *in vivo* in the PFC of VPA rats is reproduced *in vitro* by neurons grown in the absence of glia, supporting the idea of neuronal priming (Traetta et al., 2021). Cortical neurons isolated from VPA animals show a greater number of SYN and PSD-95 synaptic clusters, indicating an increase in the number of glutamatergic synapses *in vitro*. In line with this notion, it has been reported that prenatal exposure to VPA enhances the differentiation of glutamatergic synapses during cortical development (Kim et al., 2017). Our results show that prenatal exposure to VPA primes cortical neurons from VPA animals and that these cells have the intrinsic capability to mimic *in vitro* the synaptic changes seen *in vivo* in the PFC of VPA animals. Moreover, neurons from VPA animals develop a more complex dendritic arbor. In accordance with these results, Schafer et al. (2019) have shown that cortical neurons obtained from iPSC from patients with ASD show altered chronology differentiation and greater dendritic complexity.

Recently, impairments in synaptic pruning have been proposed as the main trigger for ASD. Genetic manipulation of microglia during development proved to lead to phenotypes resembling ASD core symptoms (Zhan et al., 2014; Filipello et al., 2018). Particularly, in our study, although isolated microglia from VPA animals exhibit a mild pro-inflammatory profile *in vitro*, at PND3, microglia from VPA animals display a typical morphology of early stages of development (Ueno et al., 2013). However, juvenile VPA rats show a higher proportion of unramified cells in the PFC, suggesting microglial activation. This chronology of microgliosis *in vivo* is in accordance with what was observed in patients with ASD, where microgliosis is not evident at an early age but appears throughout development (Lee et al., 2017). Moreover, the reactive profile of microglia of juvenile VPA animals in cortical areas agrees with that in other reports in VPA animals (Bronzuoli et al., 2018) and in patients with ASD (Morgan et al., 2010; Tetreault et al., 2012; Suzuki et al., 2013).

Our *in vitro* findings imply long-lasting changes in cortical microglia toward a pro-inflammatory profile when grown in the absence of neurons. Cortical microglia isolated from VPA animals when synaptic changes have already occurred (PND3) exhibit a reactive profile in culture conditions, similar to what occurs *in vivo* in juvenile stages. Besides, mild pro-inflammatory microglia from VPA animals still respond to a pro-inflammatory stimulus (LPS) but show resistance to a phagocytic challenge. These alterations could result from epigenetic alterations produced by VPA exposure directly on microglia since microglia colonization begins around E10, and elevated protein synthesis in these cells leads to autism-like behavior (Xu et al., 2020). However, in the hippocampus, where there are no obvious synaptic alterations at an early age, microglia isolated from VPA animals are not affected (Traetta et al., 2021). Indeed, the microglia phenotype could be induced by the abnormal neuronal microenvironment (Marshall et al., 2014; Lenz and Nelson, 2018). In line with this hypothesis, findings in patients suggest that glial alterations are associated with neuronal alterations (Morgan et al., 2012). Likewise, when the microglia from VPA animals are grown *in vitro*, devoid of neuronal factors during its differentiation, they show a reactive morphology, suggesting that the abnormal environment before isolation may have a long-term impact on the *in vitro* culture.

Interestingly, neuron-microglia co-cultures show that cortical microglia isolated from VPA animals are adapted to their neuronal milieu. In the presence of neurons isolated from VPA animals, microglia from VPA animals show a non-reactive morphological profile, similar to that of the neuron-microglia co-cultures obtained from control animals. Besides, microglia isolated from VPA animals mimic the neurite outgrowth effects of control microglia (Zhang and Fedoroff, 1996) regardless of the neuronal substrate. However, microglia isolated from control animals acquire a different morphological profile when exposed to neurons isolated from VPA animals by promoting dendritic shortening and reducing the dendritic tree. These findings suggest that microglia from control animals can differentiate between the VPA and control neuronal substrates, while cortical microglia from VPA animals are insensitive or adapted to their neuronal substrate. These results highlight the incapability of microglia from VPA animals to restore synaptic alterations since they are unable to differentiate neuronal substrates and respond to a phagocytic stimulus (synaptosomes). In fact, microglia are essential for refining circuits (Stevens et al., 2007) and inhibiting excessive cortical transmission (Badimon et al., 2020). This microglial adaptation to the neuronal milieu may contribute to the absence of microgliosis in the PFC of VPA animals at PND3.

An intriguing result is that although microglia isolated from VPA animals seem adapted to neuronal cues, microgliosis has been consistently described in the PFC of juvenile VPA animals along with astrogliosis (Codagnone et al., 2015; Bronzuoli et al., 2018). Interestingly, astrocytes acquire adult morphology during postnatal development (Farhy-Tselnicker and Allen, 2018). Indeed, at the neonatal period, the PFC of both control and VPA animals evidence radial structures and very few astrocytes with mature morphology. However, at the juvenile stage, robust astrogliosis is found in the PFC of VPA animals (Codagnone et al., 2015; Bronzuoli et al., 2018), which is consistent with findings in cortical areas in patients with ASD (Laurence and Fatemi, 2005; Vargas et al., 2005; Fatemi et al., 2008). Our *in vitro* findings disclose that cortical astrocytes isolated from VPA animals display a reactive morphological pattern along with a pro-inflammatory cytokine profile. Moreover, astrocytes from VPA animals show an imbalanced miRNA processing machinery, suggesting an extensive alteration in the regulation of the pro-inflammatory gene expression program. It is worth mentioning that dysregulation of miRNA expression machinery has been associated with pro-inflammatory profiles in different diseases of the central nervous system (Sun et al., 2019). Consequently, cortical astrocytes from VPA animals evidence long-lasting changes when differentiated *in vitro* in the absence of neuronal signals. Remarkably, astrocyte maturation in the cerebral cortex depends on vGLUT-1-expressing synapses (Morel et al., 2014). As this glutamate transporter is increased in the PFC of VPA rats at PND3, such neuronal change could impact astrocytes during postnatal maturation. In agreement with our findings, Voineagu et al. (2011) have shown that the astrocytic changes observed in patients are not related to genetic alterations associated with ASD, suggesting that they are a consequence of neuronal alterations or environmental events.

In addition, we show that the presence of astrocytes enables microglia from VPA animals to display a pro-inflammatory profile similar to that observed *in vivo* at PND35. In turn, microglia from VPA animals also exacerbate astrocyte reactivity. Furthermore, microglia-astroglia interaction does not allow the engagement of microglia in response to phagocytic stimuli. These results suggest that cortical microglia from VPA animals in the presence of astrocytes are not able to counteract changes in synapse number. It is known that bidirectional communication between microglia and astrocytes plays a key role in neuroinflammation (Zhang et al., 2010; Matta et al., 2019; Matejuk and Ransohoff, 2020). Herein, we provide evidence of a distinctive microglia-astroglia crosstalk in VPA animals responsible for microgliosis and exacerbation of astrogliosis. In fact, active microglia induce reactive astrocytes by secreting diverse cytokines, such as IL1, IL6, and TNFα (Zhang et al., 2010; Liddelow et al., 2017). In turn, reactive astrocytes release gliotransmitters such as ATP to trigger microglia activation (Coco et al., 2003; Bianco et al., 2005; Davalos et al., 2005) and cytokines that may modulate microglia inflammatory profile (Kettenmann et al., 2013). Microglia and astroglia from VPA animals showed a particular cytokine expression profile and a profound alteration of the miRNA processing machinery that may reveal a complex altered microglia-astroglia crosstalk in the PFC of VPA animals. It has been shown that microglia-astrocyte interaction is crucial for the proper synaptic development of the thalamus and spinal cord (Vainchtein et al., 2018). Thus, future studies of this crosstalk may reveal interesting targets that could be modulated.

To sum up, our findings are the first to show *in vitro* long-lasting changes in cortical microglia and astroglia induced by prenatal exposure to VPA. Cortical microglia from VPA animals are insensitive or adapted to the neuronal milieu and incapable of responding to phagocytic stimuli. Microglia-astroglia communication in VPA animals enables a microglial pro-inflammatory program and exacerbates astrogliosis without engaging microglia in response to phagocytic stimuli. Thus, we provide evidence that cortical microglia are not able to counteract synaptic changes and that microglia-astroglia crosstalk is a key player in neuroinflammation observed in experimental models and patients with ASD. Our study highlights cortical microglia-astroglia communication as a new mechanism implicated in neuroinflammation in ASD; consequently, we propose this crosstalk as a potential target for interventions in this disorder.

## Data Availability

The original contributions presented in the study are included in the article/Supplementary Material; further inquiries can be directed to the corresponding author.

## Abbreviations

ASD, autism spectrum disorder; ATP, adenosine triphosphate; DAPI, 4′,6-diamidino-2-phenylindole dihydrochloride; DIV, days *in vitro*; DMEM, Dulbecco’s Modified Eagle medium; E, embryonic day; FCS, fetal calf serum; GAD-67, glutamic acid decarboxylase 67; GFAP, glial fibrillary acidic protein; Iba1, ionized calcium-binding adapter molecule 1; IF, immunofluorescence; IL1β, interleukin-1β; IL6, interleukin-6; LPS, lipopolysaccharide; MAP-2, microtubule-associated protein 2; miRNA, microRNA; PND, postnatal day; PSD-95, postsynaptic density protein-95; PFC, prefrontal cortex; RT-PCR, reverse transcription polymerase chain reaction; SD, standard deviation; ST, synaptosomes or synaptic terminals; SYN, synaptophysin; TEM, transmission electron microscopy; TNFα, tumor necrosis factor α; vGLUT-1, vesicular glutamate transporter 1; VPA, valproic acid.
