# Supplementary material for: Long-Lasting Changes in Glial Cells Isolated From Rats Subjected to the Valproic Acid Model of Autism Spectrum Disorder
Source: Front Pharmacol. 2021 Aug 5;12:707859. doi: 10.3389/fphar.2021.707859 (PMC8374432; doi:10.3389/fphar.2021.707859)
Supplement: Supplementary file 1 [file DataSheet1.PDF]

Supplementary Material

1 Supplementary Methods

Behavioral assessment and analysis

Male Wistar rats used for immunofluorescence assays and transmission electron microscopy studies at postnatal day (PND) 35, as well as male siblings of the animals used for primary cultures were behaviorally evaluated to validate the VPA model. We analyzed 8 control animals from 6 different dams and 7 VPA animals from 4 different dams. During early development, animals were studied using the negative geotaxis test between PND7 and 10 to determine maturation of the vestibular reflex and motor development as previously described (1). The test consisted of placing the animal with its head down on a 25° inclined plane and the parameter measured was the time (latency) that it took to make a 180° turn (2). The average of three trials per animal was calculated and 180 s was established as the maximum time on the platform for each attempt. Between PND12-15, eye opening was evaluated by assigning scores: 0, 1 or 2, according to the number of eyes open at the day of observation (1).

At the juvenile stage (PND30-35), social preference was assessed using the three-chamber test (3). The apparatus consisted of three chambers connected to each other by two openings [each compartment: 20 cm x 40 cm (length x width)]. Prior to the test, the animal was habituated in the central compartment for 5 min. In the first 10-minute session of the test, the connections between chambers were opened and the animal was allowed to freely explore the environment to assess preference for a particular compartment. In case of preference for any compartment, the animal was excluded from subsequent analysis. In the next 10-minute session, the animal explored the three chambers, but in this case, an empty cage was placed in one of the chambers and an identical cage containing an unfamiliar naïve animal was placed in the opposite chamber. The time that the animal remained in each chamber was determined. The sociability index was defined as: (Time in the chamber with the animal - time in the chamber with the object) / (Time in the chamber with the animal + time in the chamber with the object).

References

1. Codagnone MG, Podestá MF, Uccelli NA, Reinés A. Differential Local Connectivity and Neuroinflammation Profiles in the Medial Prefrontal Cortex and Hippocampus in the Valproic Acid Rat Model of Autism. Dev Neurosci. 2015;37(3):215–31.

2. Altman J, Sudarshan K. Postnatal development of locomotion in the laboratory rat. Animal Behaviour. 1975 Nov 1;23:896–920.

3. Moy SS, Nadler JJ, Perez A, Barbaro RP, Johns JM, Magnuson TR, et al. Sociability and preference for social novelty in five inbred strains: an approach to assess autistic-like behavior in mice. Genes Brain Behav. 2004 Oct;3(5):287–302.

2 Supplementary Figures

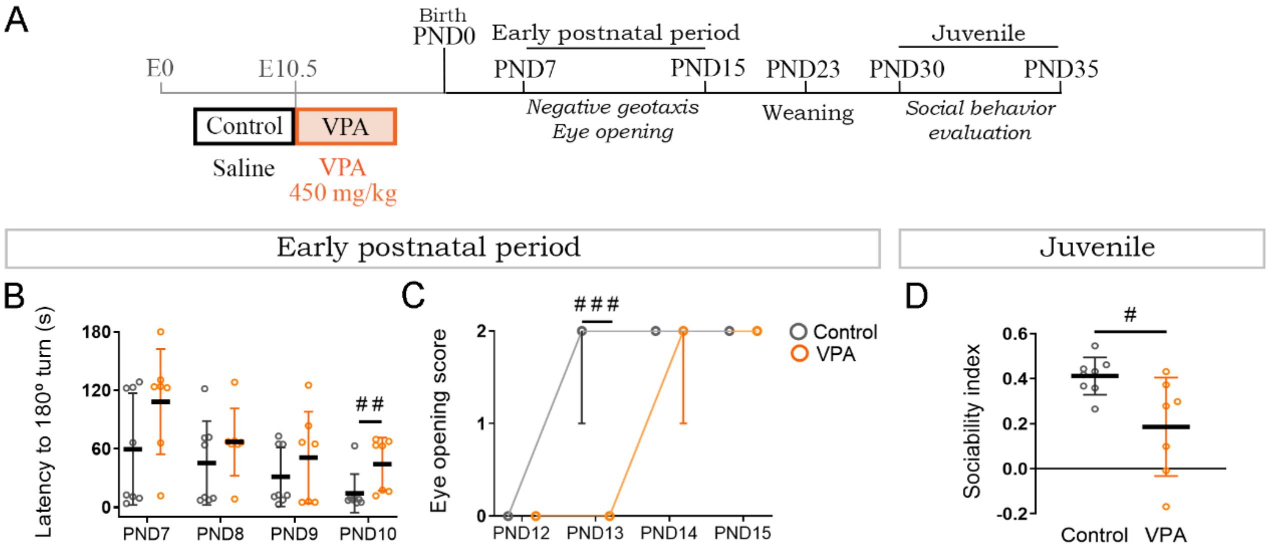

Supplementary Figure 1. Altered development and impairment of social behavior in VPA animals. A) Prenatal VPA exposure determined experimental groups. Pups were evaluated during the early postnatal period when negative geotaxis and eye opening were assessed and at the juvenile stage when the social behavior was tested. **B)** VPA animals showed a poor performance in the negative geotaxis test, being statistically significant at PND10. **C)** VPA animals opened their eyes later than controls. **D)** Impairment in social behavior was evidenced by the 3-chamber test where VPA animals got a lower sociability index defined as: (Time in the chamber with the animal - time in the chamber with the object) / (Time in the chamber with the animal + time in the chamber with the object). **B and D:** Results are expressed as mean values (± SD; control n= 8 animals, VPA n= 7 animals) **C:** Results are expressed as median with interquartile range (control n= 8 animals, VPA n= 7 animals) #p<0.05, ##p<0.01, ###p<0.001 between groups by Mann-Whitney U Test.

3 Supplementary Tables

Supplementary Table 1. Statistic (T/W), number of samples (n) and degrees of freedom (df) for the comparisons of two independent samples.

Figure		Parameter	Test	T/W	Control (n)	VPA (n)	df	p value
1	D	SYN relative immunoreactive area - PND3	Student's *t*	2,57	6	5	9	0,0302
		SYN relative immunoreactive area - PND35	Student's *t*	2,73	4	6	8	0,0258
	F	SYN puncta number/ 100 µm^2	Student's *t*	4,52	34	36	68	<0,0001
		Individual SYN puncta area	Mann-Whitney U	1402	34	36	na	0,0218
		Total SYN puncta area	Student's *t*	4,35	34	36	68	<0,0001
	H	Primary dendrite length	Student's *t*	2,44	43	43	84	0,0169
		Number of primary dendrites	Student's *t*	2,53	44	44	86	0,0131
		Number of secondary dendrites	Mann-Whitney U	1420,5	44	44	na	<0,0001
		MAP-2 area/ neuron	Student's *t*	1,21	35	35	68	0,2317
	I	SYN puncta number/ 30 µm MAP-2	Student's *t*	0,05	26	26	50	0,9627
2	B	vGLUT-1 relative immunoreactive area	Student's *t*	3,95	5	4	7	0,0055
	C	Synapse number/ µm^2	Student's *t*	2,93	4	4	6	0,0264
	E	PSD-95 puncta number/ 100 µm^2	Student's *t*	4,2	38	39	75	0,0001
		Individual PSD-95 puncta area	Mann-Whitney U	1692,5	38	39	na	0,0319
		Total PSD-95 puncta area	Student's *t*	4,2	38	39	75	0,0001
	F	GAD-67 puncta number/ 100 µm^2	Student's *t*	1,88	15	15	28	0,0712
		Individual GAD-67 puncta area	Mann-Whitney U	212	15	14	na	0,9304
		Total GAD-67 puncta area	Student's *t*	1,9	15	15	28	0,0676
3	B	Iba1 relative immunoreactive area - PND3	Student's *t*	0,78	6	5	9	0,4558
		Iba1 relative immunoreactive area - PND35	Student's *t*	0,38	5	6	9	0,7135
	C	% Ramified cells - PND3	Mann-Whitney U	30	6	5	na	>0,9999
		% Unramified cells - PND3	Mann-Whitney U	30	6	5	na	>0,9999
		% Ramified cells - PND35	Mann-Whitney U	45	5	6	na	0,0043
		% Unramified cells - PND35	Mann-Whitney U	15	5	6	na	0,0043
	D	Relative cell size	Student's *t*	1,55	2	2	2	0,1301
		Internal complexity	Student's *t*	3,13	2	2	2	0,0442
	E	Circularity	Mann-Whitney U	1934,5	42	35	na	<0,0001
		Area/ cell	Mann-Whitney U	995	42	35	na	0,0002

	**F**	IL1β	Mann-Whitney U	10	4	4	na	0,0286
		TNFα	Student's *t*	10,83	4	4	6	<0,0001
		IL6	Student's *t*	6,65	4	4	6	0,0006
	**G**	Drosha	Mann-Whitney U	10	4	4	na	0,0286
		Dicer	Mann-Whitney U	10	4	4	na	0,0286
**5**	**C**	GFAP relative immunoreactive area - PND35	Mann-Whitney U	15	5	6	na	0,0043
	**D**	Relative cell size	Mann-Whitney U	3	6	4	na	0,0333
		Internal complexity	Student's *t*	3,61	6	4	8	0,0035
	**E**	% Polygonal cells	Mann-Whitney U	2538	47	47	na	0,0206
		% Bipolar cells	Mann-Whitney U	2229	47	47	na	0,9789
		% Stellate cells	Mann-Whitney U	1901,5	47	47	na	0,0116
	**F**	IL1β	Mann-Whitney U	10	4	4	na	0,0286
		TNFα	Student's *t*	7,07	4	4	6	0,0004
		IL6	Student's *t*	65,65	4	4	6	<0,0001
	**G**	Drosha	Mann-Whitney U	26	4	4	na	0,0286
		Dicer	Student's *t*	4,62	4	4	6	0,0036
**1 Supp**	**B**	Latency PND7	Mann-Whitney U	71	8	7	na	0,0939
		Latency PND8	Mann-Whitney U	63	8	7	na	0,4634
		Latency PND9	Mann-Whitney U	60,5	8	7	na	0,6297
		Latency PND10	Mann-Whitney U	80,5	8	7	na	0,0025
	**C**	Eye opening score PND12	Mann-Whitney U	52,5	8	7	na	>0,9999
		Eye opening score PND13	Mann-Whitney U	28	8	7	na	0,0003
		Eye opening score PND14	Mann-Whitney U	48	8	7	na	0,4
		Eye opening score PND15	Mann-Whitney U	0	8	7	na	na
	**D**	Sociability index	Mann-Whitney U	37	8	7	na	0,0289

na: non-applicable

Supplementary Table 2. Statistic (H) and degrees of freedom (df) for the comparisons of more than two independent samples by Kruskal-Wallis test.

Figure		Parameter	H	df
**3**	**H**	Circularity	62,98	3
		Area/ cell	52,21	3
**4**	**B**	% Cells type I	26,03	5
		% Cells type II	33,28	5
		% Cells type III	25,59	5
		% Cells type IV	27,05	5
	**D**	Primary dendrite length	58,64	5
	**E**	Number of primary dendrites	30,12	5
	**F**	Number of secondary dendrites	19,28	5
	**G**	Dendritic tree	60,02	5
	**H**	SYN puncta number/ 100 µm2	19,79	5
	**K**	Circularity	40,29	3
		Area/ cell	21,08	3
**6**	**B**	% Polygonal cells	21,85	3
		% Bipolar cells	3	3
		% Stellate cells	26,49	3
	**D**	% Polygonal cells	11,86	3
		% Bipolar cells	1,8	3
		% Stellate cells	7,96	3
	**F**	% Ramified cells	18,93	3
		% Unramified cells	19,04	3
